# Neuralgia in the Teeth

**Published:** 1894-12

**Authors:** Thos. C. Bunting

**Affiliations:** East Mauch Chunk, Pa.


### NEURALGIA IN THE TEETH.

THOS. C. BUNTING, M. D., EAST MAUCH CHUNK, PA.

This case came under my notice last summer. A young nurse-girl subject to terrible neuralgia pains in right side of face, of which she had suffered frequent attacks for over a year. They came suddenly at night waking her from sleep—began and centered in a hollow lower bicuspid—a tearing, beating, and bursting, extending to the ear and right side of the head, causing great agony, screaming and moaning cry. A little relief was obtained by hot applications.

A peculiar symptom was the relief by burrowing or pushing the side of head into the pillow and pressing on a hard surface.

Being of the Pulsatilla temperament with corresponding symptoms, a dose of the remedy gave no relief after twenty minutes. She got one dose of Belladonna$^{200}$, which relieved at once, and after three minutes went into a long, sound sleep, from which she awoke with only a little soreness. Several days after an extensive aphtha appeared on inside of cheek and on gums about the affected teeth. Several times there was a threatened return of the pain but a dose of Belladonna promptly stopped it. This patient had been accustomed to suffer in similar attacks for a whole night under palliative treatment, and scarcely believed she was cured, except by a miracle.

